# Sprouted Grains: A Comprehensive Review

**DOI:** 10.3390/nu11020421

**Published:** 2019-02-17

**Authors:** Paolo Benincasa, Beatrice Falcinelli, Stanley Lutts, Fabio Stagnari, Angelica Galieni

**Affiliations:** 1 Department of Agricultural, Food and Environmental Sciences, University of Perugia, Borgo XX Giugno 74, 06121 Perugia, Italy; beatricefalcinelli90@gmail.com; 2 Groupe de Recherche en Physiologie végétale, Earth and Life Institute-Agronomy (ELI-A), Université catholique de Louvain, 5 (Bte 7.07.13) Place Croix du Sud, 1348 Louvain-la-Neuve, Belgium; Stanley.Lutts@uclouvain.be; 3 Faculty of Bioscience and Technologies for Food, Agriculture and Environment, University of Teramo, Via Carlo Lerici 1, 64023 Teramo, Italy; fstagnari@unite.it; 4 Council for Agricultural Research and Economics, Research Centre for Vegetable and Ornamental Crops, Via Salaria 1, 63030 Monsampolo del Tronto, Italy; angelica.galieni@crea.gov.it

**Keywords:** whole grain, germination, sprout, elicitation, phytochemical, health, microbiological safety

## Abstract

In the last decade, there has been an increase in the use of sprouted grains in human diet and a parallel increase in the scientific literature dealing with their nutritional traits and phytochemical contents. This review examines the physiological and biochemical changes during the germination process, and the effects on final sprout composition in terms of macro- and micro-nutrients and bioactive compounds. The main factors affecting sprout composition are taken into consideration: genotype, environmental conditions experimented by the mother plant, germination conditions. In particular, the review deepens the recent knowledge on the possible elicitation factors useful for increasing the phytochemical contents. Microbiological risks and post-harvest technologies are also evaluated, and a brief summary is given of some important in vivo studies matching with the use of grain sprouts in the diet. All the species belonging to *Poaceae* (*Gramineae*) family as well as pseudocereals species are included.

## 1. Introduction

Nowadays consumer lifestyle has shift towards “healthy living and healthier foods”, consequently food demand is more oriented towards diets rich in fruits and vegetables, characterized by high content of bioactive molecules. A particular role is played by ready-to-eat vegetables harvested at the initial and very earliest plant growth stages, which are commonly known as sprouted seeds. The term “sprouted seeds” involves different types of products obtained from seeds, depending on the part of the plant collected and consumed—in particular whether the seed is comprised or removed—and on the growing substrate and environmental conditions during sprouting. For each of these products several ambiguous commercial definitions occur (i.e., microgreens, shoots, babygreens, cress, wheatgrass), widespread even in the scientific literature, and the same term could refer to different types of product.

This frequently leads to misunderstanding, depriving specialists of the basic terminology on which it is necessary to point out, since the only legal definition in Western countries is given for “sprouts” and “sprouted grains”.

“Sprouts” (Regulation (EC) No 208/2013) are “the product obtained from the germination of seeds and their development in water or another medium, harvested before the development of true leaves and which is intended to be eaten whole, including the seed”. “Sprouted grains” are defined by the American Association of Cereal Chemists (AACC) with the endorsement of the United States Department of Agriculture (USDA) as follows: “malted or sprouted grains containing all of the original bran, germ, and endosperm shall be considered whole grains as long as sprout growth does not exceed kernel length and nutrient values have not diminished. These grains should be labelled as malted or sprouted whole grain” [1].

Basically, it is essential to clarify that: (i) shoots are intended to come from the germination of seeds in water, to produce a green shoot with very young leaves and/or cotyledons—the final product does not include the seed teguments and the root [2,3]; (ii) cress is the shoots grown in soil or hydroponic substrate, sold as the entire plants [2,3]; (iii) microgreens is the most common and equivocal market term, it may be referred to as seedlings at fully expanded cotyledon stage [4], or at the first true leaf stage, sold with stem, cotyledons, and first true leaves [5]; (iv) wheatgrass is the youngest stage of wheat plant which grows from the wheat grain and takes 6–10 days to germinate [6]. In general, wheatgrass refers to shoots of *Triticum aestivum* Linn. [7] but it could be extended to other species of *Triticum* genus as well as to species from *Poaceae* family, although several authors have utilized specific terms such as “ricegrass” [8] and “barleygrass” [9]. For this reason, the generic term “cereal grass” could be utilized. In this review we mainly consider sprouted seeds (sprouts and cereal grass) obtained from whole grains of the *Poaceae* family, including the *Triticeae* (barley, rye, wheat and triticale), *Oryzeae* (rice varieties), *Aveneae* (oats), and *Andropogoneae* (sorghum and maize), as well as minor grains such as millet [10]. Pseudocereals such as quinoa, amaranth, and buckwheat are also considered whole grains, as they are nutritionally similar to the *Poaceae* family [11].

### 1.1. Use of Sprouted Seeds in Human Nutrition

Sprouting of seeds has been known for a very long time, mainly in the Eastern countries where seedlings are traditionally consumed as an important component of culinary history. Starting from the 1980s, the consumption of sprouted seeds raised popularity also in the Western countries due to the consumer demand for dietetics and exotic healthy foods; in the latest years the interest around sprouted seeds has been focusing principally on low processing and additive-free. Given their peculiar characteristics such as unique color, rich flavor and appreciable content of bioactive substances, they could be used to enhance the sensorial properties of salads, or to garnish a wide variety of high-quality products [5]. Moreover, sprouting is a simple and inexpensive process which can be done without sophisticated equipment, has a quick production cycle (two to three weeks at most), occupies very little space in greenhouse production [12,13] and provides fairly high yields [14].

Beside malting—which represents a special kind of germination used for the production of alcoholic beverages—cereal seedlings might be consumed in the form of ready-to-eat sprouts or further processed, e.g., dried or roasted [15]. A possible trend is the supplementation of wheat bread in flour from sprouted cereals and pseudocereals [16]. However, the high accumulations of enzymatic activity under uncontrolled germination conditions may adversely affect the physical properties of dough and the resulting baking performance, making the use of sprouted cereals for baking more challenging [17]. The dehydrated sprouted cereals can be also used for making noodles, pasta, laddu, unleavened bread and porridge [18]. Functional beverages—obtained by lactic acid fermentation of mixture based on sprouted grains and flour [19]—represent a possible future perspective. Indeed, cereals contain water-soluble fiber, oligosaccharides and resistant starch, and thus have been suggested to fulfill the probiotic formulations. At least, wheatgrass is mostly consumed as fresh juice or as tablets, capsules and liquid concentrates [20]. Further perspectives could be given by the use of cereal sprouts as supplements in animal feeding, as it has been proposed for non-grain species [21,22].

## 2. Changing in Chemical Composition during Germination

By definition, germination incorporates those events that begin with the uptake of water by the quiescent dry seed and terminate with the elongation of the embryo axis, usually the radicle, which extends to penetrate the structures that surround it [23]. The subsequent mobilization of the major storage reserves is associated with the growth of seedling [23]. Therefore, physical and biochemical events underlie this process, i.e., weakening of seed covers, turning on of metabolic activity, activation of gene transcription, relaxation of the embryonic cell walls, and reassembly and biogenesis of organelles [24].

Briefly, during a first phase (Phase I) there is a rapid imbibition of water by the dry seeds until all of the matrices and cell contents are fully hydrated. Then, a second phase (Phase II) involves a limited water uptake (plateau phase) but a strong metabolic reactivation. The increase in water uptake associated with Phase III is associated with cell elongation leading to completion of germination [23]. Upon imbibition, the quiescent dry seeds rapidly restore their metabolic activity, including remobilization, degradation and accumulation, which imply important biochemical, nutritional and sensorial changes in the edible products [25]. The outcoming primary and secondary metabolites exert differential biological health effects when consumed compared with non-germinated seeds [11,26,27].

The extent of the changes of the principal metabolites, observed during whole grains germination, as well as the involved enzymatic activity are reported along this section. However, it should be underlined that most of the reported results refer to seedlings observed in the early germination stages and strictly depend on the species, seedling growth stage, germination conditions and laboratory techniques, which can greatly differ among experiments.

### 2.1. Carbohydrates

#### 2.1.1. Non-Structural Carbohydrates

Since one of the most studied processes on seedling development is the mobilization of complex polymers, such as starch, the substantial alterations in grain carbohydrates have been extensively studied in most of the sprouted grains [28,29].

In germinating grains, amylases catalyze the hydrolysis of starch, stored as amylose and amylopectin, to simple sugars, i.e., the reducing sugars glucose and maltose and, to a lesser extent, the non-reducing sugar sucrose [28], resulting in a higher digestibility [30,31].

However, trends in sugar profile of sprouted grains mainly depend on species: rice, sorghum, and millet seem to accumulate more maltose than glucose, whilst buckwheat greatly accumulates glucose instead of maltose [32,33]. 5-day old buckwheat sprouts showed a glucose to maltose ratio of 3.5:1 at germination temperature of 20 °C [32]; this ratio seemed to be linked to the levels of *α*-amylase and *β*-amylase released in cereals during malting [33]. Differential effects have also been reported in response to germination time, with sucrose as the dominant source of carbohydrate during early wheat germination phase and glucose and maltose during the later stages (3 days post-imbibition) [28]; also in rice, sucrose predominated shortly after imbibition. However, germinated rice seedlings showed high sucrose levels for a long post-imbibition period, glucose content increased more rapidly, and maltose did not appear at a significant level until much later (7 days post-imbibition) [34]. Sprouted grain tissue also plays a significant role: higher glucose content was located in the endosperm of wheat and rice, compared with sucrose in the scutellum [28,34]. However, the dynamics in the transport mechanism for uptake of remobilized carbohydrate reserves across the scutellum from the endosperm slightly vary among these two species.

At the very least, the dynamics of starch hydrolysis are related to starch granule type and location [11] and to the starch components. In general, the hydrolysis of starch results in little changes in amylose content, as observed in several species, including wheat [35].

#### 2.1.2. Structural Carbohydrates

Dietary fibers represent an important component of the whole grain. Cellulose, hemicellulose, and lignans are water insoluble fibers, while *β*-glucans and arabinoxylans (AXs) are grouped as water-soluble dietary fiber [15] although they are either water extractable or water unextractable [36]. Barley (5–11%) and oats (3–7%) are particularly rich in *β*-glucans [37], as well as sorghum and millet [38], while other cereals species contain only lower amounts.

The effect of germination on dietary fiber content of sprouted grains is often inconsistent and strictly depends on fiber fraction, germination time and genotypes [11]. In germinated wheat the increase in total dietary fibers seemed appreciable at 196 h of incubation whilst their content within the first 48 h of germination time even decreased [39,40]. No significant effects were recorded for barley when sprouted for 72 h [41]. In rice, an increase in total dietary fiber after malting has been observed and could be explained with the formation of new primary cell walls [42]. Besides the increase in total fiber content, an upward trend has also been recorded for both soluble and insoluble fiber fractions of germinated brown rice varieties, while the ratio soluble vs. insoluble ones varied in response to genotype and processing conditions [43]. Rising in the insoluble fraction during germination has also been observed for oats [37].

In sprouted barley and oats the hydrolytic activity of endogenous *β*-glucanases determined a significant reduction in *β*-glucans [15]. This leaded to a decrease in the total soluble fibers over time, up to 144 h [37], and to a different behavior with respect to species characterized by lower *β*-glucans content, such as wheat, where the amount of soluble fraction remained constant up to 96 h and then increased steadily until 168 h [39].

The extent of changes in AXs content during germination has garnered much less attention than *β*-glucan. AXs are non-starch polysaccharides, found as cell wall constituents and esterified with ferulic and p-coumaric acids. In general, the total AXs content is not significantly affected by sprouting [38]. However, significant lower content in AXs has been associated with the germination of oats and rye [44]; during malting of barley, Han [45] observed a loss of up to 50% of total AXs. On the other hand, the amount of water extractable AXs can be increased during sprouting by the release of soluble AXs previously covalently bound to other cell walls, resulting in altered proportions of total to water extractable AXs [46,47].

### 2.2. Proteins

Whole grains major storage proteins of most cereals are classified according to their solubility properties into albumins (water-soluble), globulins (salt-soluble), glutelins (alkali-soluble), and prolamins (alcohol-soluble). During grain germination, the storage proteins are hydrolyzed into peptides and amino acids by proteolytic enzymes after 2–3 days from imbibition, thereby increasing nutrient bioavailability [48].

It is well known that prolamins content decreases as time of sprouting increases, as observed in triticale, barley, rye, oats [14] and wheat [39]. On the other hand, several authors have reported an increase in crude proteins in barley [49], waxy wheat [40], brown rice [50] and oats [51], as a result of the proteins and amino acids stored in the cereal being decomposed by water absorption, changed into transportable amides, and supplied to the growing parts of the seedlings [52]. However, the protein content depends on the balance between protein degradation and protein biosynthesis during germination [53].

Besides an increase in amino acids contents [33,40], significant alterations in free amino acid composition have been observed. In particular, germinated whole grains contain higher quantities of essential amino acids, which take part in protein production in human body. Grain type and germination time have the greatest influence on the amino acid composition. In waxy wheat, the essential amino acids isoleucine, leucine, phenylalanine and valine reached maximum levels after 36 h of germination, while other essential amino acids (i.e., threonine and methionine) were highest after 24 and 48 h [40]. Rice and buckwheat malts produced higher amounts of amino acids after 5 and 4 days of germination, respectively; both species were characterized by very low amounts of asparagine, methionine and histidine [32]. In oats, an increase in albumin content (rich in essential amino acids lysine and tryptophane) and a subsequent decrease in globuline and prolamine contents (poor in lysine) have been observed during germination [54].

#### 2.2.1. *γ*-Aminobutyric Acid

*γ*-Aminobutyric acid (GABA) is a four-carbon non-protein amino acid that is produced primarily by the *α*-decarboxylation of L-glutamic acid, catalyzed by glutamate decarboxylase (GAD). It acts as the main inhibitory neurotransmitter in the mammalian cortex [55]. As far as cereals are concerned, GABA production has been deeply studied in brown rice, although several researchers have observed a variation in GABA content also during germination of wheat and barley [40,56]. Regardless of species, GABA dramatically increases during sprouting. In brown rice, GABA content in seedlings was enhanced by 2- to 5-fold depending on varieties [57], up to 8- to 12-fold when sprouted for 2 to 4 days at 27 to 35 °C [58,59], supported by the increased GAD activity and reduced glutamate content during germination [60]. However, GABA content in cereal seedling is greatly affected by both environmental conditions during sprouting (i.e., temperature or abiotic stress) and grain treatment before germination (i.e,. steeping or soaking).

### 2.3. Lipids

Even in cereals, where starch is the main carbon store in the endosperm, lipids are abundant in the living tissues of whole grains (i.e., embryo, scutellum and aleurone) as oil (triacylglycerols, TAG). The mobilization of TAG from oil bodies requires a coordinated metabolic activity, which is started on with germination, leading to the net conversion of oil to sugars [61]. The lipases firstly release the esterified fatty acids (FAs) from TAG. Free fatty acids (FFAs) can then be degraded through the *β*-oxidation and glyoxylate cycles and subsequently converted into sugars [61]. Further information on the pathways involved in the conversion of TAG into sugars in germinated seeds can be obtained from the reported literature.

In oats—which is unique among cereals due to its high content in oil as compared to starch and protein concentration—the degradation of oil reserves from the embryo was observed to take place earlier than that of oil reserves in the scutellum [62]; the mobilization of TAG reserves in the endosperm started later, 1–2 days after imbibition, and coincided with the accumulation of FFAs in this tissue [62]. In waxy wheat, sprouting did not significantly affect FA composition of both free and bound lipids as well as the content of essential FAs (linoleic and linolenic acids) during 48 h of germination [40]. Sprouting had a significant effect on FAs composition of 9-day old wheat seedlings, as observed by Ozturk et al. [63]: the linolenic acid (18:3 n3) content increased, while the amount of cis-18:1 and cis,cis-18:2 FAs decreased. Conversely, after 3 days of germination the FAs more represented in wheat sprouts were palmitic acid, linoleic acid and oleic acid [64].

In addition, in this case it is important to point out that the metabolic dynamics of FAs is strictly dependent on the pre-germination treatments and lipase activity in whole grain tissues, and levels of some individual FAs either progressively increased or decreased over germination time.

The *γ*-oryzanol is the principal component of unsaponificable lipid fraction that, along with tocopherols and tocotrienols, contributes to the nutritive characteristics and health promoting properties associated to rice seedlings [65]. After germination, the *γ*-oryzanol content in rough rice and brown rice increased by 1.13 and 1.20-fold, respectively [66]. However, the methods that maximize the content of *γ*-oryzanol from rice seedlings could be further investigated, since its concentration depends on cultivar-specific content in kernels [67], rates of water uptake during sprouting and germination timing [65].

### 2.4. Phytate and Minerals

Phytate is highly concentrated in several food items derived from plants; it represents the major storage form of phosphorus in mature grains and legumes [68]. However, in human beings the insufficient endogenous intestinal phytase limits phosphorus utilization [69]; phytate also negatively impacts the bioavailability of mineral ions such as Zn^2+^, Fe^2+/3+^, Ca^2+^, Mg^2+^, Mn^2+^ and Cu^2+^ since it is characterized by a strong chelation affinity with cations and is therefore considered an antinutritional factor [68].

Phytases are a subfamily of the high-molecular-weight histidine acid phosphatases, involved in the hydrolysis of phytate to myo-inositol and ortho-phosphate, as well as inorganic phosphate. The phytase activity tends to increase during germination; in barley, Sung et al. [70] found a very low phytase activity at the beginning of sprouting which, in the first couple of days, increased to 8-fold. However, the concentration of phytase in whole grains greatly varies among cereal species, with rye having the highest values and oats the lowest ones [15]. As a consequence, phytate content diminishes to a different extent during germination (see also [69]). Sprouting of brown rice over a period of 12–72 h leaded to a reduction of 60% in phytate content [71], whereas in 4-day old sorghum seedlings, a degradation of up to 87% of phytate was observed [72]. Further studies on phytate degradation during germination processes have been reported for brown rice [50], barley [37], pearl millet [69,73], corn [74], sorghum and wheat [69].

As phytate content decreases, bioavailability of phosphorus and minerals increases. Germination induced a decreasing Ca concentration in barley and wheat [75] and an increasing Mg concentration in barley, oats [37], and wheat [75]. In corn, the content of the main macro-elements (Na, K, Mg, Ca and P) tended to decrease after 2 days of germination and then to increase up to 6 days; trace minerals (Fe, Zn, Mn, Cu and Co) showed a clear upward trend over germination time [74]. For these species, an increase in HCl-extractability of both major- and trace- minerals was also observed during sprouting, despite it was greatly affected by cultivar [74]. Ca, Fe and Zn extractability increased from 76.9, 18.1 and 65.3% in the whole grain to 90.2, 37.3% and 85.8%, respectively, after 96h of germination in finger millet [76]. Lemmens et al. [77] observed an increase in Zn and Fe bio-accessibility from 15 and 14% in sprouted wheat to 27 and 37% in hydrothermally processed sprouted wheat, respectively.

### 2.5. Antioxidants

Whole grains contain high concentrations of antioxidants, such as polyphenols, carotenoids, ascorbic acid and tocopherols, which balance oxidative damages of seedling cell components [78].

Phenolic acids in seeds are present in both free and bound fractions, with the latter as the most representative, linked to hydrolyzable tannins, lignins, cellulose and proteins, which are mainly structural components of bran and aleurone [79]. Generally, germination leads to a mild increase of the total polyphenols content, although different contribution from free and bound fractions is observed, depending on species and sprouting conditions [80,81]. The free fraction increases and the bound one decreases as sprouting goes on, as observed in 2-day old wheat, grown under controlled conditions [82], as well as in 12-day old seedlings of emmer and einkorn cultivars [83]. Conversely, in waxy wheat the bound fraction content dropped after 12 and 24 h of sprouting and then significantly increased after 36 and 48 h [40]; similar trend was reported for several tetraploid and hexaploid *Triticum* species [79,83,84], although sprouts examined in these researches were characterized by different growth stages. Indeed, ferulic and *p*-coumaric acids, which greatly contribute to the total bound fraction, are known to be involved in cell wall structure and development [85]. Conversely, in brown rice both free- and bound- fractions significantly increased over germination time [86]; this is probably due to the hydrolysis of conjugated phenolic compounds and to contemporaneous de novo biosynthesis in the embryo axis [87]. Tartary buckwheat is naturally rich in flavonoids such as rutin, quercetin and catechin; in 8-day sprouts, high rutin and catechin and lower quercetin concentrations were observed with respect to whole grains [88]. Interestingly, oats is unique among cereals containing avenanthramides (AVAs)—i.e., low molecular weight soluble phenolic compounds—which was reported to increase by approximately 20% during germination [15].

The germination process allowed an increase of *β*-carotene in wheat [89], while barley malts showed limited increases or decreases, depending on the variety [90].

Vitamin C content in grains is generally very low; however, many studies have reported higher vitamin C levels in germinated barley [91] and wheat [75,89], probably due to its de novo synthesis [38]. Moreover, in wheat—which contains all tocopherol forms—the contents of *α*-, *β*-+*γ*- and *δ*-tocopherols were significantly affected by sprouting and increased by 3.59-fold, 2.33-fold and 2.61-fold, respectively [87]. A significant higher content of tocopherol was also reported for germinated rice [42], where it plays an important role in limiting non-enzymatic lipid oxidation during seedling development.

## 3. Factors Influencing Nutritional Quality of Germinated Whole Grains

### 3.1. Genotype and Seed Source

The most significant role in the determination of nutritional value of sprouted grains is played by the genotype. In the last years, several studies focused on the characterization of grains from different ancient and modern cereals genotypes [92,93,94,95,96,97] as well as pseudocereals species [98,99], principally in terms of bioactive compounds.

As is known, the biochemical composition of whole grains is also conditioned by the environmental condition during crop growth, especially during grain development. Bellato et al. [100] have investigated on the content of total polyphenols, antiradical activity and 5-n alkylresorcinols of 30 Italian commercial varieties of durum wheat, grown in two different geographical areas located in Central and Southern Italy. Results showed that the contribution of genotype (G) x environment (E) interaction to the total variability was lower than that due to the separate effects (G and E), and E accounted for the highest proportion of the variation. In particular, alkylresorcinols concentration increased in environment with dry conditions during grain filling, while high water availability during grain development was favorable to free phenols accumulation. Environment has been also indicated as the main factor contributing to the total variation in some quality parameters in the case of winter and spring wheat varieties [101]. In the same location, phenolic and flavonoid contents in durum and soft wheat were higher in years characterized by lower temperatures and higher rainfall during the 30 days before harvest [102]. Different effects of abiotic stresses during ripening (i.e., drought or high temperatures) have been observed [103], with increases in protein [104,105] and carotenoid content [106], and contrasting effects on starch quality [107]. This is generally because dehydration stress shortens the grain filling period.

The nutritional value of wheat grains is also influenced by the exposure of the mother plant to biotic stress (i.e., pathogens, weeds) and nutrient shortage. Many studies, focusing on the differences in nutritional values between organic and conventional cereals, have led to contradictory results [108]. In general, organic products have a lower content of proteins; however, old wheat varieties show a more efficient nutrient use in low-N environments compared to the modern ones, which are strictly dependent on high-level of available N [109]. Although phenolic compounds accumulate more under biotic stress conditions, and consequently organic crops are often thought to contain more phenolic compounds, the effect of cultivation system on secondary metabolites content is often not significant, also in terms of single phenolic acids [108]. The influence of different agronomic practices or environmental stresses on secondary metabolites in the field is naturally modified by the effects of other potential co-variables. Under greenhouse conditions, three Chilean landraces of quinoa exposed to two levels of salinity (100 and 300 mM NaCl) starting from 34 days after sowing showed a deep change in the amino acid composition and protein profiles of the main seed storage proteins as well as in the contents of bioactive molecules [110].

### 3.2. Germination Conditions

Biochemical changes during sprouting occur depending on germination conditions as well as on the “seed invigoration” treatments applied to the grains in order to improve the germination and post-germination seedling growth. Seed priming is a pre-sowing treatment during which seeds are hydrated with a solution that allows them to imbibe and go through the first reversible stage of germination but does not allow radicle protrusion through the seed coat [111]. Common priming techniques include osmopriming (soaking seeds in osmotic solutions such as polyethylene glycol, PEG), halopriming (soaking seeds in salt solutions) and hydropriming (soaking seeds in water) [111].

Recently, researches were addressed to identify the optimal combination of temperature and time during pre-sowing and sprouting treatments, to obtain higher quality sprouts, especially in terms of phytochemical content. These goals are achieved through the use of sophisticated statistical techniques including the response surface methodology approach. For example, in Ecuadorian brown rice cultivars, soaked grains (deionized water, 28 °C, 24 h) were introduced in a germination cabinet at 28 and 34 °C in darkness for 48 and 96 h [112]. The multiple linear regression predicted optimal germination conditions for accumulation of GABA and antioxidant activity after soaking followed by germination at 34 °C for 96h, while the highest total phenolic content was obtained in the combination of 28 °C for 96 h, although differences between genotypes were recorded [112]. In foxtail millet the highest total phenolic content, total flavonoid content and antioxidant activity were obtained with 15.84 h of soaking in tap water at room temperature and 40 h of germination at 25 °C [113]. The optimum germination conditions for sorghum suitable for supplementary food formulations (i.e., low tannin and high protein contents) were established to be steeping for 24 h at 31 °C plus germination for 4.5 days at 30 °C [114]. The highest antioxidant concentrations in wheat sprouts were achieved following 7 days of germination at 16.5 °C [89] while in purple corn sprouts it was possible to maximize the content of GABA, total phenolic compounds and antioxidant activity using a germination temperature of 26 °C for 63 h [115]. The same conditions were guaranteed by kiwicha (Amaranthus caudatus) sprouts richer in GABA and phenolic compounds [116], whilst the highest phenolic content in sprouted quinoa was obtained at 20 °C for 42 h [117]. The results reported here refer to specific experiments conducted in controlled environments, under specific laboratory conditions and germination times, so that the stage of cereal grass is not always reached. Accordingly, the optimization of seedling growth parameters needs to be deeply investigated (see also Section 2).

Not always pre-sowing treatments induce a greater accumulation of bioactive compounds. During the 24 h soaking period, an increase of total phenolic compounds was observed in brown rice [118] and wheat [89]. The pH of the soaking solution can affect the enzymatic activities in seeds allowing to enhance or reduce the phytochemical content in sprouted grains. In brown rice the optimal pH for higher GABA content ranged from 3.0 to 5.8 [119], since a lower cytosolic pH stimulates GAD activity [120]. GABA content of germinated barley grains after steeping in a buffer solution (pH 6.0) was slightly higher than that in water [56].

Sub-optimal conditions during germination process can lead to an accumulation of phytochemicals in seedlings due to the activation of secondary metabolism [121]. Rehydration involves high levels of oxidative stress, so that abiotic stress induced during seed germination can intensify the production of reactive oxygen species (ROS), which could damage the structures of DNA, protein, lipid, and other macromolecules in the seeds. Therefore, ROS scavenging is pivotal for seed germination under stress conditions and comprises non-enzymatic components, mainly linked with overproduction of antioxidants (e.g., phenolics) [122]; an induced environmental stress during germination can be classified as abiotic elicitor.

It is important to highlight that the stressful conditions during sprouting, as well as the pre-sowing grain treatments, may reduce germination percentage and/or dry matter production. It follows that the commercial use of those manipulations of environmental conditions during germination should be appropriately set up for each species, to obtain sprouts characterized by higher nutritive and health promoting values, without or slightly affecting the production levels.

#### 3.2.1. High and Low Temperatures

Few studies investigated on the effects of extreme temperatures applied during germination process on the nutritional quality of sprouted seeds; most of these researches focused on non-gramineous species like for example lentil [123,124], broccoli [125], alfalfa and radish [126]. In soft white winter and dark northern spring wheats, the GABA content increased during 48 h of germination and after sequential hydration with anaerobic and heat treatments [127]; additionally, in waxy hull-less barley, steeping in water at 5 °C significantly enhanced GABA content in 72 h germinated sprouts [56]. Cold stress has been also reported to regulate the biosynthesis of anthocyanins in various plants, including 12-day old tartary buckwheat seedlings, grown for 4 days at 4 °C [128]. Stressed buckwheat sprouts enhanced anthocyanin contents, especially in the epidermal cells, so removing ROS and decreasing the osmotic potential of cells [129]. Furthermore, anthocyanin-rich sprouts display brighter colors, which help improve their nutritional and health profiles attracting more consumers.

#### 3.2.2. Light Modulation

Light is one of the main factors affecting plant growth and development. Photoreception systems respond to light intensity and quality as well as to light duration and intermittence, thus determining plant morphogenetic changes, functioning of the photosynthetic apparatus, and trend of metabolic pathways [130]. Moreover, lighting conditions might evoke the photoxidative changes in plants, which lead to an altered action of the antioxidant defense system [130]. Despite the effects of the light irradiance level are well documented, data regarding the effect of light spectral quality in plant metabolism are still limited.

As far as sprout production is concerned, the use of light emitting diodes (LEDs) during seeds germination seems to be the most relevant strategy to improve the nutritional quality of seedlings. LEDs are characterized by wavelength specificity and are available in the spectral range from near ultraviolet (UV) to near infrared (IR). Thus, light spectra can be obtained through the selection of specific wavelengths, also in combination to each other, to maximize the accumulation of specific compounds [131]. Inherently, some studies have evaluated the effects of different LEDs treatments on carbon-nitrogen metabolism and pigments concentration. Urbonavičiūtė et al. [132] compared wheatgrass and barleygrass (7–9 cm height) grown under control (high pressure sodium lamps, HPS) and light treatments (HPS supplemented with amber LEDs, 595 nm). Supplementary flashing amber light induced an increase by 1.6 and 1.3 times of fructose and glucose content in wheat seedlings, while enhancing the content of glucose and the concentration of xanthophylls cycle pigments (i.e., neoxanthin, violaxanthin and zeaxanthin) in barley [132]. In seedlings (14-day old) of two rice varieties (purple and green leaf), blue light increased Chl a/b ratio, Chl fluorescence and total proteins in leaves, while the content of anthocyanin in seedling leaves was highest in red + blue LEDs [133]. Similarly, in barley seedlings grown under red + blue LEDs the amino acids content of plants was twice compared to control plants grown under sun-light [134]. The richest accumulation of total carotenoids in tartary buckwheat sprouts was observed under white light than under blue and red light [135].

The impact of single wavelength under different lighting conditions on radical scavenging activity, total phenol content and interactions with other antioxidants during seed germination remains still unclear. This is probably due to the seed sensitivity that is strongly conditioned by genetically determined amount of antioxidant compounds found in the tissues.

Supplementary red light enhanced the content of phenolic compounds in seedlings of several gramineous and non-gramineous species [130,136]. Red LED also increased the content of γ-tocopherol in 15-day old barley sprouts [137], and the total phenolic content [138] and catechin content [139] in buckwheat sprouts. The maximum rutin concentration in buckwheat sprouts was obtained after 4 days from sowing either by red:green:blue (4:1:1) LEDs combination [140] or blue light [139]. Again in buckwheat seedlings, blue light contributed also to the highest total anthocyanin content [141]. On the other hand, supplementary amber light (595 nm) did not significantly affect phenolic and vitamin C contents in wheat and barley sprouts [132], while supplemental green light caused a significant increase in α-tocopherol in 3-day old wheat seedlings [130]. The effect of UV-B (>300 nm) irradiation was effective in the production of buckwheat sprouts with increased anthocyanins, rutin and DPPH (2,2-diphenyl-1-picryl-hydrazyl-hydrate) radical scavenging activity [142].

The time interval of exposure to natural light periods have been investigated in common and tartary buckwheat, obtaining highest vitamin C, rutin and free amino acids contents with increasing natural light periods with respect to growing under complete darkness [143]. However, total phenolic and flavonoid contents were greater in 7 days-old sprouts of buckwheat grown in dark chamber than in 7 days-old sprouts grown in open field under natural environment [144]. Nevertheless, in the former study the natural light periods were not continuous throughout the sprouting periods, while in the latter the germination conditions were different for other variables (i.e., substrate and temperatures). Moreover, longer growth (14-day old seedlings) under light significantly increased rutin and total flavonoid contents compared to darkness-grown sprouts [145]. This could be explained by the upregulation of some structural genes involved in flavonoid biosynthesis under light, as observed in 2, 4, and 6-day old tartary buckwheat sprouts grown in light/dark environment compared to dark-treated ones [146].

Finally, it is worth to notice that, besides the effects on sprout composition, the light intensity, spectrum and duration affect the energy consumption [147] and thus the economic cost of indoor growing, which is the most common situation in sprout production.

#### 3.2.3. Salt Stress

Salinity causes one of the most important abiotic stresses in plants, especially during the early seedling growth, which is a very salt-sensitive phase. However, to date, little is known about the effect of salinity on the phytochemical accumulation in edible sprouts since the impact of salt stress has been principally evaluated in terms of germination rates and physiology. Studies on grains are limited, so that an accurate analysis of the current literature over this topic needs to include evidences from some non-gramineous species.

In buckwheat sprouts, the amount of phenolic compounds, in particular isoorientin, orientin, rutin, and vitexin, as well as carotenoids increased with NaCl solution treatments (10, 50, 100, and 200 mM), regardless of the growth stage [148]. In einkorn, an increase in total bound-phenolics fraction, until 50 mM NaCl solution was observed, due to the induction of p-coumaric and trans-ferulic acids in their bound form, while durum wheat coped with salinity by increasing the total free-phenolics and reducing the bound ones [149]. Also, the total phenolic content, reducing power, superoxide radical scavenging and thiobarbituric acid reactive substances production inhibition were measured in raw and denatured aqueous extracts from sprouts and wheatgrass of einkorn and emmer at increasing salinity levels [150]. For these genotypes the best compromise between quality traits (increased with increasing salinity levels) and growth (reduced at increasing salinity) was 25–50 mM NaCl, i.e., the salinity level which maximized the polyphenol and antioxidant yield [150].

In foxtail millet, NaCl stress enhanced GABA content, the associated GAD activity and free amino acids concentration, which acted as a substrate for GABA accumulation [151]. The optimal GABA accumulation in germinated tartary buckwheat under salinity was obtained from 5-day old seedlings in 34 mM NaCl solution [152].

#### 3.2.4. Hypoxia Stress

The alteration of gas composition during germination has been investigated in several species. In particular, a GABA accumulation in seedlings, in response to hypoxia stress during sprouting, was observed in soybean [153,154], faba bean [155] and brown rice [156]. The stimulation of GABA synthesis is an adaptive response of plant tissues to stress-induced cytosolic acidosis, associated to the oxygen deficit stress [157]. Indeed, hypoxia stress is often investigated in combination to acidic culture solutions as germination substrates. The maximum GABA content in sprouted foxtail millet under hypoxia was obtained with a germination temperature of 33 °C, an air flow rate of 1.9 L/min and a pH solution of 5.8, with citrate buffer solution (10 mmol/L) being more effective than acetate one [158]. In germinated tartary buckwheat the optimization of GABA content was reached through the following germination conditions: culture temperature 31.25 °C, air flow rate 1.04 L/min and pH 4.21 (citric acid buffer—10 mmol/L) [159].

#### 3.2.5. Other Elicitors

Several other abiotic and biotic elicitors as well as plant hormones have been evaluated as potential factors for improved nutritional quality. In general, literature data refer to different elicitation treatments applied to the same species, also in combination to each other, thus highlighting the species-specific response to elicitors. Treatments applied to non-gramineous species, reported in this section, could be usefully transferred also to gramineous ones, properly modulating the experimental conditions (i.e., concentrations and application time). Moreover, all the reported “pre-harvest” elicitation treatments referred here either as seed priming treatments or germination in water solution contain the elicitors, as well as spraying elicitors solution on cotyledons. Therefore, deeper investigations on standardized and species-specific elicitation protocols are still needed.

Among abiotic elicitors, the oxidative elicitation (20 mM and 200 mM hydrogen peroxide - H_2_O_2_ - solutions) was studied by Świeca [160] on 8-day old lentil sprouts. The stressful condition, imposed from 2 days after sowing, gave the highest amounts of phenolics in seedling tissues, especially in terms of chlorogenic, ferulic, o-coumaric and salicylic acids, as well as in terms of ability to protect lipids against peroxidation, which approximately raised up 12-fold and 8-fold. In 3-day old quinoa sprouts, treatments with H_2_O_2_ (5 mL of 50 mM and 200 mM H_2_O_2_ solutions sprayed on 1-day-old sprouts) were evaluated also in combination with solutions of phenolic precursors i.e., shikimic acid, L-phenylalanine and L-tyrosine (0.1 mM) [161]. Oxidative stress significantly enhanced total polyphenols and flavonoids contents, whilst the phenylpropanoid pathway feedings were effective only in flavonoids accumulation; all the investigated treatments significantly improved antioxidant capacity, phenylalanine ammonia-lyase (PAL) and tyrosine ammonia-lyase (TAL) activities, although to different extents, depending on elicitors combinations [161].

Inorganic salts and metal ions can be considered as abiotic chemical elicitors [162]. The use of selenium, as sodium selenite or sodium selenate, has been found to increase the content of seleno-proteins and the soluble conjugated forms of phenolic acids in rice [163], so improving the antioxidant defense system; moreover, selenium promotes the increase of glutathione peroxidase and superoxide dismutase activities [164]. In germinated wheat seeds, the application of sodium selenite solutions (5–10 ppm) in the textile germination beds, positively affected the vitamin C content and the antioxidant activity of wheat seedlings [165]. In amaranth sprouts the effect of sodium selenite on total reducing capacity and antioxidant activity was strictly related to the genotype and no correlation between selenium concentration and glutathione peroxidase activity was found [164].

The application of artificially produced mineral-rich water for sprouts cultivation did not influence the content and composition of dietary fiber in wheat seedlings, while significantly decreased total dietary fiber and catechin and increased quercetin in tartary buckwheat [88]. At the same time, trace element water (provided by Shimanishi Kaken Co., at the concentrations of 100, 200, 300, 400 and 500 ppm), as spraying treatments (4 h intervals) during germination, did not affect rutin, quercitrin and quercetin contents in buckwheat sprouts (about 5–7 cm length), while 300 ppm trace element water induced higher radical scavenging, ion chelating and superoxide anion scavenging activities [166]. Treatments at different concentrations of Al^3+^, Cu^2+^, and Zn^2+^ (metallic additives) induced higher contents of both total flavonoids and D-chiro-inositol in sprouted tartary buckwheat [167]. In germinated brown rice, the effects of electrolyzed oxidizing water (pH, 3.0; oxidation reduction potential, 1079.0 mV) were effective in inhibiting microbial growth during germination as well as in enriching GABA content in seedlings by 1.7-fold on average [168].

Among biotic elicitors, polysaccharides and oligosaccharides application during germination has been widely investigated. In particular, the effect of chitosan has been investigated in several non-gramineous species such as bean [169], soybean [170,171,172], lentil [173], broccoli [174] and lettuce [175]. Additionally, yeast polysaccharide (YPS) has been regarded as an efficient biotic elicitor for stimulating secondary metabolite production. In tartary buckwheat, YPS applied during germination (50, 100, 200, 400 and 800 mg/L) stimulated flavonoids production in 10 days-old sprouts [176], while four fungal polysaccharides—obtained from the endophytic fungus Bionectra pityrodes Fat6—increased total rutin and quercetin contents, via the stimulation of the phenylpropanoid pathway [177]. Exogenous elicitation with yeast extracts of Saccharomyces cerevisiae (0.1% *w*/*v*) had little or no significant influence on total phenolic content and antioxidant activity of sprouted wheat [25], while significantly increased the levels of ferulic, p-coumaric and syringic acids in broccoli sprouts [178]. Other investigated biotic elicitors include mannitol [179], sucrose [180,181,182], glucose [180,181,183], glucosamine and collagen [184] as well as tea tree [175] and Salix daphnoides bark [25] extracts. Moreover, among protein elicitors, fish protein hydrolysates (FPH)—produced from byproduct of fishery industry—seem interesting due to their high content of proline and proline precursors. In particular, FPH solutions (0, 5, 10, 15 and 20 mg/L), applied to rice grains during soaking for 24 h at room temperature (28 ± 2 °C), stimulated phenolic contents in ricegrass via proline-linked pentose phosphate and shikimate pathways [185].

Supplying some biosynthetic precursors, as well as specific phytohormones, during germination may enhance biosynthesis of secondary metabolites. Several studies have been conducted on species belonging to Brassicaceae family, applying exogenous elicitors such as methionine, tryptophan, jasmonic acid, salicylic acid (SA) and methyl jasmonate (MeJA), either as soaking solution treatment [174] or directly sprayed on sprouts [180,186,187], in order to evaluate their effects on glucosinolate and polyphenols. Elicitation with MeJA (0.1 mM MeJA dissolved in 0.25% ethanol—sprayed on sprouts) increased total polyphenols (+54.2%) and flavonoids (+61.5%) just as isoorientin, orientin, rutin, and vitexin by about 18% also in 7-day old buckwheat sprouts [188]. Similar results are reported by Kim et al. [189], who observed significant increases in phenolics due to the stimulation of the phenylpropanoid pathway by MeJA treatments. In buckwheat sprouts, phenolics content were also increased by elicitation with tyrosine (+30%) and shikimic acid (+17%) [161].

Treatments with pulse electric fields (PEF) could potentially be used to manipulate seed imbibition and germination, resulting in seedlings with different metabolite compositions. Grains of Triticum aestivum L. appropriately hydrated (water content of 45% or greater) and subsequently electrostimulated by PEF (electropriming), significantly increased glutathione levels and the activities of several enzymes associated with antioxidant metabolism in plant cells, in 7-day old seedlings [190].

### 3.3. Biofortification

Germinated whole grains would be a promising vehicle for food biofortification programs. In brown rice, a recent research by Wei et al. [191] has shown the possibility to increase Fe concentration in germinated grains (24 h of sprouting) by soaking kernels in solutions of FeSO_4_, just before germination process. Fe fortification increased Fe concentration of 1.1–15.6 times in brown rice sprouts, due to the penetration of Fe solutions across the aleurone layers via the dorsal vascular bundle present in the endosperm [192], as well as Fe solubility, which was nearly 4-fold higher than in non-fortified sprouts [191]. However, the relatively low permeability of some seed coats does not allow obtain fortification with Fe enriched solutions. This was the case for broccoli and radish soaked with Fe(III)-EDTA and Fe(III)-citrate solutions [193]. Conversely, the same authors observed significantly higher iron concentrations in 5-day old alfalfa sprouts obtained from Fe-soaked seeds. This increase was associated with a significant decrease in Ca, Mg, Na and/or Mn concentrations, due to the leakage during imbibition, and with a significant induction of phenolic compounds concentration [193].

Plant seeds are able to accumulate Se and to transform it from inorganic to organic form (i.e., Se-containing proteins) during germination. In this way, Se-biofortification during sprouting could represent a valid strategy to improve Se concentration in seedlings [163]. In 3-day old tartary buckwheat sprouts, the total Se concentration tended to rise up with increasing external selenite treatments [194]. In brown rice, both total Se and protein-bound Se contents in seedlings significantly increased with increasing external selenite concentration (up to 60 μmol/L Na_2_SeO_3_) and germination time (4-day old sprouts); moreover, germination time promoted the transformation of inorganic Se to protein-bound Se, despite a very non-uniform distribution in Se-containing proteins was observed [195]. In wheat, Se enriched kernels in combination with some enzymatic and performance traits (i.e., α-amylase activity) can be obtained with 35 mg/L Na_2_SeO_3_ in germination medium for 24 h at 25 °C [196].

Fortification programs can also be applied during crop cycles, representing a suitable approach to ameliorate the concentration of macro- and micro-elements in whole grains, thus influencing their dynamics during the subsequent germination process. Foliar Zn fertilization, applied over panicle initiation and grain filling stages, represents an effective agronomic practice to promote rice grains Zn concentration, especially when supplied as Zn-amino acid and ZnSO_4_ [197]. Application of urea containing Zn increased Zn and protein levels in maize grains, showing better results in poor Zn soil [198]. Both foliar spray and soil application of Se significantly increased Se uptake in common buckwheat, with Se content in grains showing the highest correlation coefficient with soil Se application treatments [199].

## 4. Post-Harvest Storage and Processing Effects on Sprouts Safety and Quality

Sprouts are commonly harvested and freshly consumed, since they are naturally characterized by a rapid quality loss at relatively low temperature. As a consequence, there is the need to optimize the storage conditions through temperature control and modified atmosphere or active packaging which allow to finely manage the chemical composition of the package headspace during their shelf life. At the same time, sprouts consumption has been involved in several foodborne outbreaks due to the lack of a post-germination kill step. Many studies have been conducted over these topics even though little has been found on cereal and pseudo-cereal sprouts. Anyway, the technologies used on other species are based on common assumptions, although the genotypic variation in biochemical composition of grains may need further optimization protocols.

It is important to specify that “microgreens” in their commercial definition (see also Section 1) do not include the consumption of radicles, so that they are considered as fresh cut vegetables, and microbiological risks can be managed with the existing technologies and packaging, prior and during commercialization [200] (see also Reference [201]). It follows that the “Microbiological safety” section refers principally to the freshly consumption of sprouts (including seeds) (see also Reference [2]).

### 4.1. Microbiological Safety

Microbiological contamination of sprouts can be attributable to many potential pre-harvest and post-harvest sources of contaminants, which include seed material, germination medium and soaking water as well as transport, handling and storage of seedlings. It follows that the production process should be optimized starting from seeds treatments. All the employed strategies to reduce health risks include physical, biological and chemical applications, which are well-detailed and reviewed in Yang et al. [202] and Ding et al. [203].

Physical intervention methods include high and low temperatures, high pressure, irradiation and supercritical carbon dioxide, while biological interventions concern antagonistic microorganisms and antimicrobial metabolites. Among chemical interventions there are disinfectants and sanitizers such as ozone and chlorine, as well as electrolyzed water. 

Chlorine (50–200 ppm concentration) is widely used as the primary post-harvest disinfectant because of its broad antimicrobial activity [204,205]. It can be utilized also as dioxide gas treatment, as demonstrated by studies on mungbean sprouts artificially inoculated by Salmonella which was better controlled than by aqueous chlorine wash in reducing microbiological contamination [206]. These differences could be attributable to the capability of chlorine dioxide gas to penetrate and inactivate cells that are attached to inaccessible sites and/or are within biofilms on the sprouts surface [206]. In addition, since chlorine compounds can be inactivated by organic materials present on fresh products and may form various carcinogenic organochlorine compounds, most liquid solutions of organic acids such as lactic, acetic and malic acids have been introduced as alternatives to control pathogenic microorganisms. For example, in soybean sprouts, the application of lactic acid (*v/v*, 0%, 1.5%, 2% and 2.5%) combined with mild heat (20 °C, 40 °C and 50 °C) for 3 minutes against Shiga toxin producing Escherichia coli (O157:H7 and non-O157 serogroups including O103, O111, O145 and O26) revealed the interesting reductions of all serotypes after immersing sprouts into 2.5% lactic acid at 50 °C [204].

In common buckwheat sprouts, Chun and Song [207] have evaluated the effects on the microbial quality of the combined treatments with aqueous chlorine dioxide (ClO_2_), fumaric acid and ultraviolet-C (UV-C). Authors found that 100 ppm aqueous ClO_2_, 0.31% fumaric acid and 1.9 kJ/m^2^ UV-C represented the optimal combination condition providing reductions on the populations of total aerobic bacteria, yeast and mould, and coliform, and also maintaining an acceptable sensory quality. The combinate effect of a sanitizer mixture (100 mg/L aqueous ClO_2_ and 0.3% fumaric acid), UV-C (2 kJ/m^2^) and modified atmosphere packaging (air and CO_2_ gas) on buckwheat sprouts stored for 8 day at 4 °C, also reduced the initial inoculated counts of Escherichia coli O157:H7 and Salmonella typhimurium, without affecting color parameters [208].

An environmentally-friendly and cost-effective technology to reduce microbiological risks associated to wheat grains, contaminated with the food pathogen Listeria monocytogenes and the microfungus Botrytis cinerea, was represented by photoactivated chlorophyllin-chitosan complex (Chl-KCHS) [209].

Other techniques to ensure the hygienic safety of sprouts could include ionizing radiation treatment, electron beam (e-beam) or gamma ray irradiation [210,211], as well as their combination with sodium hypochlorite [212]. However, these techniques are subjected to limitations and are not approved in all countries; in addition, major drawbacks are represented by their detrimental effects on different seed growth parameters.

### 4.2. Longer Shelf Life

Low storage temperatures generally curtails the rates of respiration, senescence, and growth of spoilage microorganisms allowing to reduce quality loss [4,213]. However, the selection of optimum storage temperature plays a key role, depending on species-specific sensibility to chilling and natural decay rates. For example, Berba and Uchanski [200] investigated on the respiration rates and shelf life of arugula, radish, and red cabbage seedlings (without radicles), observing a higher shelf life, based on visual analysis, in radish than in the other species, either at 4 °C (21 vs. 14 days on average) or at 10 °C (14 vs. 7 days on average) storage temperatures. Anyway, their level of acceptance also in relation to quality traits (biochemical composition) also needs to be considered [214].

Of course, the effect of storage temperatures must be evaluated in combination with the atmospheric composition during packaging and with washing treatments on sprouts before packaging, which synergistically contribute to the shelf life (hurdle technology). For example, modified atmosphere packaging effectively prolongs the seedlings shelf life by decreasing O_2_ and increasing CO_2_ partial pressures in the package headspace [215]. However, factors influencing package atmosphere include product respiration rate, packaging film oxygen transmission rate (OTR), product weight, package surface area, and storage temperatures [216]. Since package size and product weight are often pre-determined in food supply chains, the selection of a packaging film with suitable OTR could represent a suitable way to maintain quality and extend shelf life [217]. For example, buckwheat sprouts (without radicles) stored at 5 °C in 16.6 pmol/s/m^2^/Pa ORT films performed optimally until 21 days of storage, maintaining highest quality attributes and tissue integrity [218]. Authors have underlined the importance of optimizing also washing (i.e., chlorine wash solutions) and drying parameters prior to storage, which could affect the microbial populations during storage [218].

In addition to storage conditions, seedlings cooling can also have an impact on post-harvest storage, as observed in mung bean sprouts [219]. Goyal and Siddiqui [220] evaluated the effects of UV- irradiation (10 kJ/m^2^ in laminar flow chamber for 1 h), pulsed electric field (50 Hz, 10,000 V pulses for 10 s), hot water dip (50 °C for 2 min) and ethanol vapors (in a glass chamber saturated with ethanol vapors for 1 h) on the quality and storage life of mung bean sprouts. Authors have concluded that both ethanol and hot water dip treatments inhibited decay development on the surface, without affecting color and sensory quality during storage (120 h at 7 ± 1 °C); moreover, hot water dip increased the total soluble solids and acidity of bean sprouts [220]. Lu et al. [221] investigated on the effects of preharvest CaCl_2_ and post-harvest UV-B radiation applications on storage quality of broccoli microgreens highlighting a greater influence of CaCl_2_ spray treatment in terms of both glucosinolates content and longer storage life. Indeed, Ca treatments are known to contribute to delay leaf senescence by cross linking with pectin polymers in cell wall and protecting cell membrane integrity [4].

At the very least, the effect of light exposure on quality and phytochemical concentrations of freshly sprouts could be considered, since they are usually displayed under light in grocery stores. Indeed, light exposure could cause detrimental effects on products quality. For example, in radish seedlings (without radicles) light exposure (continues fluorescent light; intensity of about 30 µmol/m^2^/s) accelerated quality deterioration and weight loss, while dark storage (intensity of about 0.1 µmol/m^2^/s) helped preserve quality, prolong shelf life and maintain higher levels of β-carotene and lutein/zeaxanthin ratio during 16 days of storage. On the other hand, no significant differences in α-tocopherol concentration, total phenolics concentration and antioxidant capacity were observed [222].

### 4.3. Processing

Beside the freshly consumption, sprouts can be extracted for juices or dried for soluble powder and enriched flour productions (see also Section 1.1).

During powder production the drying process plays the most important role to preserve the biochemical quality of wheatgrass. Different drying methods can be used including hot air oven, microwave and vacuum oven dryings [223]. In general, the quantitative analysis of antioxidant component of microwave dried wheat grass powder (600 W, 15 min) showed the highest mean value of total phenol content, chlorophyll and scavenging ability [223]. However, seedlings, like all foodstuff rich in phytochemicals, should be dried at low temperature: different drying processes have been proposed and investigated for wheatgrass juice [224]. Powders from wheatgrass juice can be encapsulated to avoid the undesirable odor and protect the phytochemicals, using maltodextrin and whey protein [225].

Fermentation is a widespread technique for cereal and pseudo-cereals grass processing, in order to better preserve or promote nutritionally interesting compounds [226].

Some studies have considered the effect of sprouts addition in the formulation of different products focusing on their technological properties. However, food technologies are not examined in detail here, because beyond the scope of this review. We just mention the rheological and baking properties of wheat flour fortified with fermented/germinated cowpea flour [227], the functional properties of wheat flour and the shelf-life of bread supplemented with sprouted wheat [228], the higher total sugar content of breads from recipes containing sprouts [17] as well as the higher phenolic content and antioxidant activity of enriched breads with wheat sprout powder [16], the quality characteristics of bakery products following the addition of dried barley sprout powder [229], the cooking of germinated brown rice [230,231], the size and acceptability of tortillas obtained from sprouted wheat flour [232,233]. Some rheological characteristics such as elasticity, water absorption index and viscosity of doughs supplemented with sprouts derivatives could be negatively affected and imply complications and higher production costs.

## 5. Sprouted Seeds and Human Health

A detailed review of the chemical modifications occurring during germination has been given in Section 2. Many of those modifications behave like the human digestion process [234] and implicate an improved availability of macro- and micro-nutrients as confirmed by several in vitro and in vivo studies [11,232]. Therefore, sprouted grains are a complex food matrix, where nutrients are nearly fully available, rich in various antioxidant and bioactive compounds, representing health promoting food.

In the past many health benefits have been argued by motivating the strong in vitro antioxidant potential, although the relationship between those data and the redox status measurable in vivo is very weak. Furthermore, this approach ignores other biological effects, that perhaps could be related to compounds which may not be detected by an in vitro assay, but, thanks to a good bioavailability, can activate/deactivate an oxidative-related metabolic pathway or interfere with gene regulation or with other signaling pathways, etc. [235]. In this chapter only studies that deal with the bioavailability concept will be considered.

Nowadays, the most important preclinical and clinical studies have been focused on germinated brown rice (which represents the majority of references shown in this review); anyway, a recent more in-depth review focusing on health benefits of sprouted grains, including also other cereal species, is provided by Lemmens et al. [38].

Germinated brown rice has proven strong potentials for better glycemic control, correction of dyslipidemia, amelioration of oxidative stress, reduced type 1 tissue plasminogen activator inhibitor (PAI-1), enhanced adiponectin concentration, and increased sodium potassium adenosine triphosphatase and homocysteine thiolactonase activities, which are reviewed in detail in Imam et al. [236] and Nelson et al. [11]. These effects cannot be attributable to a single individual bioactive compound, but rather to a synergic interaction between all the bioactive compounds induced also by the germination process (i.e., GABA, oryzanol, phenolics, dietary fibers and others), so obtaining a greater functional effect when consumed as a whole food [237]. The effects of white rice, brown rice and germinated brown rice in the dietary management of cardiovascular diseases have been recently investigated by Imam et al. [238] who highlighted the modulation of the lipid metabolism and oxidative stress in rats. In pregnant rats the exposure to high-fat diet plus germinated brown rice until 4 weeks post-delivery, influenced metabolic outcomes in offspring of rats with underlying epigenetic changes and transcriptional implications, that led to improved glucose homeostasis and reduced the risk of insulin resistance manifestations [239]. Germinated brown rice supplied in the diet also reduced obesity complications in high-fat diet induced-obese rats, through the improvement of lipid profiles and reduction of leptin level and white adipose tissue mass [240]. Germinated brown rice enhanced insulin levels, insulin receptor, glucose transporters and glucose metabolism in induced-hyperglycemia mice [241]. In addition, in human SH-SY5Y cells, a neuro-protective effect by gene modulations has been found to be provided by germinated brown rice extracts [242]. These evidences represent only a preliminary indication on the potential beneficial effects of germinated brown rice, and more research is still required.

Tartary buckwheat sprouts have been used as ingredient for a new “functional” pasta, which has been proven to reduce cardiovascular risk and metabolic disorders, such as hypertension in rats [243]. Authors have shown that spontaneously hypertensive rats fed with pasta containing tartary buckwheat sprouts (30% vs. 70% durum wheat semolina) exhibited improved levels of blood pressure-related biochemical parameters, probably attributable to the higher levels of rutin and its aglycone quercetin.

The consumption of wheatgrass juice has received particular attention in the case of thalassemics, principally due to its high chlorophyll content. Patients fed with wheatgrass juice during transfusion therapy showed up to 25% reduction in transfusion volume requirement, without compromising hemoglobin levels and a 29.5% increase on the mean time interval between transfusions [20]. In addition, wheatgrass juice is currently under investigation as a possible therapy for ulcerative colitis as it is rich in apigenin, which is possibly correlated with the normalization during oxidative stresses [20].

There is room for a lot of research on this subject: overall there must be a reason why diet supplementation with wheatgrass on Drosophila melanogaster improved flies’ longevity to 58 days as compared to 52 days in control flies [6].

## 6. Conclusions and Future Perspectives

Germination leads to substantial changes in biochemical composition of whole grains: starch reserves are mobilized by the action of α-amylase, that corrodes the surface of the granule and forms pinholes; the nitrogen containing fractions shift towards oligopeptides and free amino acids, and amino acids composition changes as well; triacylglycerols start to be hydrolyzed and saturated/unsaturated fatty acids ratio rises up; the amount of anti-nutritional factors (e.g., phytate, trypsin inhibitor, tannin) decreases significantly, and bioactive compounds such as phenolics, phyrosterols, folates and GABA increase. Hence, in sprouted grains almost all nutrients are fully available and various antioxidants occur at higher concentrations, thus providing the base to define sprouts as “functional foods”.

Despite the changes that are naturally found taking place during germination process, it is possible to modulate the factors affecting sprouting, i.e., trough biotic and abiotic elicitors, in order to enhance the concentrations of some important phytochemicals in sprouts.

Further research is needed to evaluate: (i) the optimization of germination process (i.e., growth stage, germination conditions, elicitors) as a function of genotype, aimed at modulating/enhancing phytochemical contents; (ii) the pre- and post-harvest technologies to reduce microbiological risks without affecting sprout nutraceutical profiles; (iii) the actual translatability of sprout bioactive compounds to biological benefits in lifestyle-related diseases, through in vivo essays. These objectives should be achieved also taking into account the productive perspective, pursuing the goals of an innovative agri-food technology beyond home-made production.
